# Kinetostatic Modeling and Performance Analysis of Redundant-Actuated 4-PSS&S Compliant Parallel 3-DOF Micro-Rotation Mechanism

**DOI:** 10.3390/mi16060612

**Published:** 2025-05-23

**Authors:** Jun Ren, Ruihan Xiao, Yahao Lu

**Affiliations:** Hubei Key Laboratory of Modern Manufacturing Quantity Engineering, School of Mechanical Engineering, Hubei University of Technology, Wuhan 430068, China; 15797311830@163.com (R.X.); hbutlyh@163.com (Y.L.)

**Keywords:** redundant actuation, compliant parallel mechanism, kinetostatic modeling, optimal distribution of actuating force, output stiffness, parasitic axis drift, workspace

## Abstract

This paper presents a novel redundant-actuated 4-PSS&S compliant parallel micro-rotation mechanism (P represents the actuated prismatic joint and S denotes the spherical pair) with three rotational degrees of freedom. First, compliance models of the flexure spherical hinge, each branch and the whole mechanism are established using the compliance matrix method. Then, the mechanism is simplified as an equivalent spring system to establish two kinetostatic models, with their correctness validated through finite element simulations. Finally, a comparative analysis is conducted on the performance of the 3-PSS&S mechanism, non-redundant-actuated 4-PSS&S mechanism and redundant-actuated 4-PSS&S mechanism. The results show the following: ① For the 4-PSS&S mechanism, redundant actuation with optimized actuating force distribution effectively reduces the peak actuating force by up to 50% (average 40.95%), achieving an average 10.79% reduction compared to the 3-PSS&S mechanism. ② The 4-PSS&S mechanism’s output stiffness increases by 26.68% in the *θ_x_* and *θ_y_* directions and by 33.31% in the *θ_z_* direction compared to the 3-PSS&S mechanism. ③ Optimal force distribution significantly reduces the parasitic axis drift of the redundant-actuated 4-PSS&S mechanism at the constrained flexure spherical hinge S3, indicating higher motion accuracy. ④ The workspace volume of the redundant-actuated 4-PSS&S mechanism expands by 94.32% compared to the 3-PSS&S mechanism and by 372.89% compared to the non-redundant-actuated 4-PSS&S mechanism.

## 1. Introduction

In recent years, with the deepening implementation of the Industry 4.0 strategy, precision engineering has become increasingly crucial in advanced manufacturing, minimally invasive medicine, bionic robotics and other fields. For instance, in semiconductor manufacturing, wafer packaging processes require nanometer-level positioning accuracy [1]. In neurosurgery, micro-manipulation instruments must achieve multi-degree-of-freedom-precision motion within a confined 20 mm × 20 mm cranial space [2]. Such special working conditions require higher and higher performance of the equipment in terms of motion accuracy, dynamic response and motion range, etc. The compliant parallel mechanism combines the advantages of compliant mechanisms, such as no clearance, no friction and no need for assembly, with those of parallel mechanisms, such as high precision, excellent bearing capacity and small motion inertia. It has gradually become an ideal carrier for achieving precise control and is widely used in fields such as Micro-Electro-Mechanical System assembly, biomedical devices and ultra-precision machining.

In the research field of parallel mechanisms, redundant actuation (where the number of actuators exceeds the mechanism’s degrees of freedom) is sometimes adopted to optimize force distribution, thereby achieving enhanced bearing capacity, improved stiffness, higher positioning accuracy and an expanded workspace. Cui et al. [3] proposed a novel 1T2R 2-SPR-UPR cable-driven redundant-actuated parallel manipulator, and by establishing the complete homogeneous Jacobian matrix of the mechanism, they concluded that the redundant-actuated parallel manipulator has much better dexterity and stiffness. Chai et al. [4] presented a 1T2R 2UPR-2PRU parallel manipulator with actuation redundancy. The stiffness models and performance distributions of the mechanism can provide references for actual applications. Han et al. [5] designed a full-redundant drive planar 6R parallel mechanism; through a kinematic analysis of the mechanism, the results show that the fully redundant actuation parallel mechanism has high precision and a large bearing capacity. Zhu et al. [6] presented a loading device based on the redundant-actuated parallel manipulator; it was concluded that compared with the non-redundant-actuated mechanism, this mechanism has a larger regular workspace, better transmission performance and stronger loading capability. The above research shows that some performance aspects of the parallel mechanism can be improved by introducing redundant actuation. Recently, Ren et al. [7] has extended the application of redundant actuation from parallel mechanisms to compliant parallel mechanisms. The *n*-4R compliant parallel micro-motion pointing mechanism was taken as the object, and its workspace was expanded by employing redundant actuation. From these studies, it can be seen that the current application of redundant actuation is mostly focused on rigid mechanisms, especially parallel mechanisms, while it is applied less in compliant parallel mechanisms.

Kinetostatic modeling is an indispensable research tool for compliant parallel mechanisms, aimed at revealing the mapping relationship between the input and output of a mechanism. Ren et al. [8] proposed a novel class of *n*-4R compliant parallel pointing mechanisms, taking 4-4R as an example, established the kinetostatic model of the mechanism and analyzed the influence of the geometric parameter values on the mapping matrix, thereby providing theoretical guidance for structural optimization. Arredondo-Soto et al. [9] presented a 3-RRR compliant spherical parallel mechanism and conducted kinetostatic modeling. Based on the model, the parasitic displacement was evaluated, demonstrating that higher stiffness ratios lead to reduced parasitic axis drift and displacements. Lu et al. [10] proposed a novel 6-DOF 3UPS parallel manipulator with multi-fingers; based on the kinetostatic model of the mechanism, the active forces/torques and workspace performance were thoroughly analyzed. The aforementioned studies have demonstrated that a kinetostatic model not only serves as the theoretical basis for precise control of a mechanism, but also provides the foundational framework for structural optimization and kinematic performance investigations.

In 2022, Ren et al. [11] proposed a 3-PSS&S compliant parallel 3-DOF micro-turntable. A characteristic of the mechanism is that its degrees of freedom and motion patterns are not affected by the change in the number of PSS-actuated branches (as long as ≥3). In this paper, inspired by the improvement in the performance of the rigid parallel mechanism by utilizing redundant actuation, a redundant-actuated branch is introduced to construct a novel redundant-actuated 4-PSS&S compliant parallel micro-rotating mechanism on the basis of the original 3-PSS&S compliant parallel mechanism, aiming to improve the bearing capacity of the mechanism and improve problems such as insufficient actuating force, parasitic axis drift and a small workspace. The motion range of the mechanism is designed to be 0~2*π* rad × 0~0.02 rad × ±0.02 rad. The remainder of this paper is organized as follows: Section 2 introduces the structure of the mechanism and the definition of parameters. Section 3 establishes dual kinetostatic models (the input/output force–displacement and input/output displacement–displacement) via the compliance matrix method, and the correctness of the models is verified by numerical examples combined with finite element simulation. In Section 4, based on the kinetostatic model, the peak actuating force, output stiffness, parasitic axis drift and workspace are analyzed, and in a comparison with the relevant performance of the 3-PSS&S mechanism and the non-redundant-actuated 4-PSS&S mechanism, the effectiveness of redundant actuation in improving the performance of the mechanism is verified. The conclusions are presented in Section 5.

## 2. Description of Mechanism and Definition of Parameters

**Figure 1:**
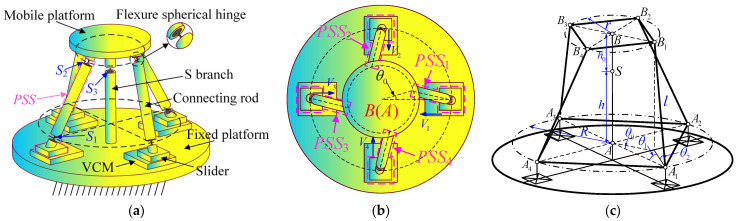
(**a**) Axonometric drawing; (**b**) top view; (**c**) kinematic sketch of mechanism.

An axonometric drawing and top view of the redundant-actuated 4-PSS&S compliant parallel mechanism are shown in Figure 1a,b, respectively. The mechanism consists of a mobile platform, a fixed platform, four actuated platforms equipped with a Voice Coil Motor (VCM), four actuated branches (denoted as PSS branches) and the constrained branch (denoted as the S branch). Four PSS branches are distributed 90° apart in the circumference direction. Each PSS branch comprises a connecting rod, a slider and two right-circular flexure spherical hinges (hereinafter referred to as “flexure spherical hinge”). The two hinges from bottom to top are denoted as *S*_1_ and *S*_2_, respectively. The lower slider is connected to the actuated platform on the base. The S branch includes a similar flexure spherical hinge (denoted as *S*_3_). The VCM actuates the slider linearly toward the S branch link. Taking into account the capacity of the actuated platform, the stroke and weight of the motor, the VCM type of TMEC-0007-004-000 is selected. Its peak output force is 6.8 N, and the total stroke is 4 mm.

In order to facilitate the kinetostatic modeling in the subsequent text, a kinematic sketch of the redundant-actuated 4-PSS&S compliant parallel mechanism is constructed, as shown in Figure 1c. *A_i_* and *B_i_* represent the geometric centers of the flexure spherical hinges *S*_1_ and *S*_2_ in each branch, respectively. Squares *A*_1_*A*_2_*A*_3_*A*_4_ and *B*_1_*B*_2_*B*_3_*B*_4_ are constructed with these points as vertices. The intersection points of the diagonals of the squares are defined as points *A* and *B*, respectively. The radii of the circumcircles of the squares are *R* and *r*, respectively. *h* and *h*_0_ represent the distances from the flexure spherical hinge *S*_3_ to the planes *A*_1_*A*_2_*A*_3_*A*_4_ and *B*_1_*B*_2_*B*_3_*B*_4_, respectively. *l* denotes the length of the connecting rod. *θ*_0_ represents the angle between the projection of *BB*_1_ on the plane *A*_1_*A*_2_*A*_3_*A*_4_ and *AA*_1_. *θ*_1_ represents the angle between the projection of the connecting rod *A*_1_*B*_1_ on the plane *A*_1_*A*_2_*A*_3_*A*_4_ and *AA*_1_. *θ*_2_ represents the angle between the connecting rod *A*_1_*B*_1_ and the plane *A*_1_*A*_2_*A*_3_*A*_4_. Based on the screw theory and the modified Grübler–Kutzbach formula, it is established that the mechanism is a 3-DOF pure rotation mechanism (the mobile platform can rotate in three directions around the geometric center of the flexible spherical hinge *S*_3_). The degree-of-freedom calculation formula is as follows:

(1) $$M=d\left(n-g-1\right)+\sum_{i=1}^{g}f_{i}+v-\zeta$$

where *M* represents the number of degrees of freedom of the mechanism; *d* represents the order of the mechanism (in the spatial mechanism, its value is 6); *n* represents the total number of components of the mechanism (one fixed platform, one mobile platform, four connecting rods and four sliders); *g* represents the total number of kinematic pairs (nine spherical pairs and four prismatic pairs); *f_i_* represents the number of degrees of freedom of the *i*-th kinematic pair; *v* represents the number of redundant constraints of the mechanism without common constraints; and *ζ* represents the number of local degrees of freedom of the mechanism (the passive rotational degrees of freedom of each connecting rod around its own axis). Therefore, degree-of-freedom of the redundant-actuated 4-PSS&S compliant parallel mechanism is:

$$M=6\times \left(10-13-1\right)+(9\times 3+4\times 1)+0-4=3$$

## 3. Kinetostatic Modeling and Verification

In this section, firstly, the compliance models of the flexure spherical hinge, each branch and the overall mechanism are established based on the compliance matrix method. Then, the mechanism is simplified into an equivalent spring system to obtain the control equation for elastic deformation. Based on this control equation, two kinetostatic models (the input/output force–displacement model and input/output displacement–displacement model) of the mechanism are derived. Finally, in combination with a spherical trajectory example, the correctness of the model is verified by comparing the theoretical calculation results with the ANSYS simulation results.

### 3.1. Compliance Modeling

In the study of the kinetostatics of a compliant mechanism, compliance modeling is a core step in kinetostatic modeling. This paper adopts the compliance matrix method. Based on the compliance matrix of compliant elements, coordinate transformation and superposition operations are performed on them, so as to construct the spatial compliance model of the overall mechanism.

#### 3.1.1. Compliance Model of the Flexible Spherical Hinge and Its Coordinate Transformation

**Figure 2:**
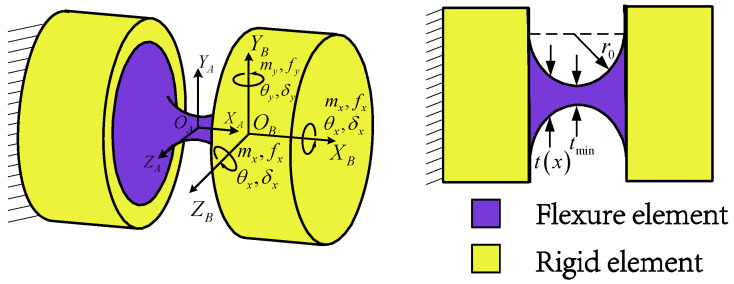
Structure of the flexure spherical hinge and coordinate system establishment.

At the beginning of compliance modeling, it is necessary to define the compliance model of the flexible spherical hinge element and its coordinate transformation. The structure and coordinate system of the flexible spherical hinge are illustrated in Figure 2, where *O_A_*-*X_A_Y_A_Z_A_* (denoted as $\left\{O_{A}\right\}$) is the local coordinate system established by the geometric center of the flexible spherical hinge *O_A_*, and *O_B_*-*X_B_Y_B_Z_B_* (denoted as $\left\{O_{B}\right\}$) is the local coordinate system established by the end-face center *O_B_* of the flexible spherical hinge *O_B_*. *r*_0_ and *t_min_* denote the cutting radius and the minimum thickness of the flexible spherical hinge, respectively.

According to Ref. [12], under the assumptions of the linear elastic theory and small deformation hypothesis, the relationship between generalized forces and generalized displacements for a single flexible spherical hinge can be expressed as follows:

(2) $$X=\left[\begin{array}{c} \theta_{x}\\ \theta_{y}\\ \theta_{z}\\ \delta_{x}\\ \delta_{y}\\ \delta_{z} \end{array}\right]=\left[\begin{array}{cccccc} C_{\theta_{x},m_{x}} & 0 & 0 & 0 & 0 & 0\\ 0 & C_{\theta_{y},m_{y}} & 0 & 0 & 0 & C_{\theta_{y},f_{z}}\\ 0 & 0 & C_{\theta_{z},m_{z}} & 0 & C_{\theta_{z},f_{y}} & 0\\ 0 & 0 & 0 & C_{\delta_{x},f_{x}} & 0 & 0\\ 0 & 0 & C_{\delta_{y},m_{z}} & 0 & C_{\delta_{y},f_{y}} & 0\\ 0 & C_{\delta_{z},m_{y}} & 0 & 0 & 0 & C_{\delta_{z},f_{z}} \end{array}\right]\left[\begin{array}{c} m_{x}\\ m_{y}\\ m_{z}\\ f_{x}\\ f_{y}\\ f_{z} \end{array}\right]=C_{B}^{O_{B}}F$$

where $C_{B}^{O_{B}}$ represents the compliance matrix of the flexible spherical hinge at the center of end face *O_B_*, and the subscripts of each element in the matrix represent the compliance coefficients in corresponding directions. The calculation methods for these compliance coefficients are detailed in Appendix A. The first three diagonal elements correspond to compliance in the principal functional directions.

To facilitate the modeling and analysis in the subsequent text, the compliance matrix is transformed from the end-face center *O_B_* to the geometric center *O_A_* using coordinate transformation, and the expression is as follows:

(3) $$C_{A}^{O_{A}}={\left[T\right]}_{O_{B}}^{O_{A}}C_{B}^{O_{B}}{\left(\left[T_{O_{B}}^{O_{A}}\right]\right)}^{T}$$

where the adjoint matrix ${\left[T\right]}_{O_{B}}^{O_{A}}$ is explicitly defined as follows:

(4) $${\left[T\right]}_{O_{B}}^{O_{A}}=\left[\begin{array}{cc} {\left[R\right]}_{O_{B}}^{O_{A}} & 0_{3\times 3}\\ {\left[D\right]}_{O_{B}}^{O_{A}}{\left[R\right]}_{O_{B}}^{O_{A}} & {\left[R\right]}_{O_{B}}^{O_{A}} \end{array}\right]$$

${\left[R\right]}_{O_{B}}^{O_{A}}$ is the rotation matrix from the coordinate system $\left\{O_{B}\right\}$ to $\left\{O_{A}\right\}$, and ${\left[D\right]}_{O_{B}}^{O_{A}}$ is the skew-symmetric matrix defined by the translation vector ${\left[d\right]}_{O_{B}}^{O_{A}}={\left[\begin{array}{ccc} x & y & z \end{array}\right]}^{T}$; it can be expressed as follows:

(5) $${\left[D\right]}_{O_{B}}^{O_{A}}=\left[\begin{array}{ccc} 0 & -z & y\\ z & 0 & -x\\ -y & x & 0 \end{array}\right]$$

#### 3.1.2. Compliance Modeling of Each Branch and the Overall Mechanism

**Figure 3:**
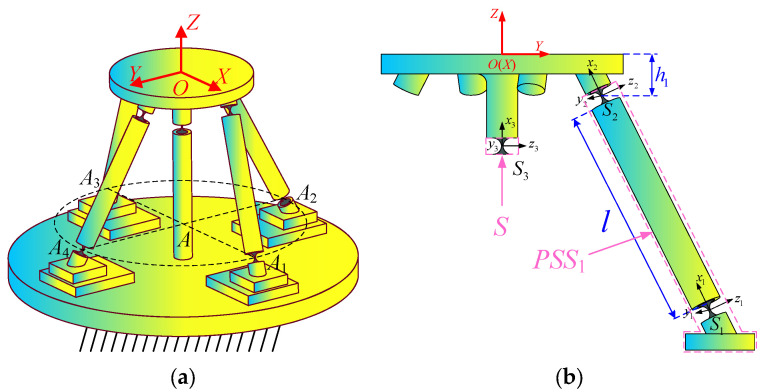
(**a**) The global coordinate system; (**b**) the compliance model of each branch.

Considering that the overall mechanism consists of four identical PSS branches and one S branch, it is only necessary to analyze the compliance of one PSS branch and the S branch, and then the overall compliance of the mechanism can be obtained through rotation and superposition. The global coordinate system *O*-*XYZ* (denoted as {*O*}) is defined to be located at the center point *O* of the mobile platform. The directions of its *X* and *Y* axes are consistent with *AA*_1_ and *AA*_4_ (as shown in Figure 1c), respectively, as shown in Figure 3a. The compliance models of the PSS_1_ branch and the S branch are constructed as shown in Figure 3b. The self-coordinate system {*S_j_*} (*j* = 1, 2, 3) is established with the geometric centers of the three flexible spherical hinges {*S_j_*}, and the *x* direction of each coordinate system is made to coincide with the direction of the connecting rod axis. The local coordinate system of the PSS_1_ branch is set at {*S*_2_}, where *h*_1_ is the vertical distance from *S*_2_ to the upper surface of the mobile platform.

As derived from Equation (3), the compliance matrices of the three flexible spherical hinges *S_j_* in their respective self-coordinate systems {*S_j_*} are expressed as follows:

(6) $$C_{S_{1}}=C_{S_{2}}=C_{S_{3}}=C_{A}^{O_{A}}$$

To compute the compliance matrices of the PSS_1_ branch, the compliance matrix of *S*_1_ in the local coordinate system {*S*_2_} can be determined through coordinate transformation. Through calculating the pose parameters between coordinate systems using mathematical modeling and applying the superposition principle from serial compliant mechanism modeling, the overall compliance of the PSS_1_ branch in {*S*_2_} is derived as

(7) $$C_{PSS_{1}}^{S_{2}}=C_{S_{1}}+C_{S_{1}}^{S_{2}}=C_{S_{1}}+{\left[T\right]}_{S_{1}}^{S_{2}}\;C_{S_{1}}{\left({\left[T\right]}_{S_{1}}^{S_{2}}\right)}^{T}$$

where

(8) $$\left\{{\left[T\right]}_{S_{1}}^{S_{2}}=\left[\begin{array}{cc} {\left[R\right]}_{S_{1}}^{S_{2}} & 0_{3\times 3}\\ {\left[D\right]}_{S_{1}}^{S_{2}}{\left[R\right]}_{S_{1}}^{S_{2}} & {\left[R\right]}_{S_{1}}^{S_{2}} \end{array}\right]\right.\;,\;{\left[R\right]}_{S_{1}}^{S_{2}}=I_{3\times 3}\;,\;{\left[d\right]}_{S_{1}}^{S_{2}}={\left[\begin{array}{ccc} -l & 0 & 0 \end{array}\right]}^{T}$$

In order to establish the overall compliance model of the mechanism, it is necessary to establish the compliance matrix $C_{PSS_{1}}^{O}$ of the PSS_1_ branch in the global coordinate system {*O*}. Considering that the four PSS branches are evenly distributed, the compliance matrices $C_{PSS_{i}}^{O}$ of the other three PSS*_i_* (*i* = 2, 3, 4) branches in the global coordinate system {*O*} can be obtained by rotating the PSS_1_ branch’s compliance matrix in {*O*} by 90°, 180° and 270° around the *Z*-axis of the global coordinate system {*O*}:

(9) $$\left\{\begin{array}{c} C_{PSS_{1}}^{O}={\left[T\right]}_{S_{2}}^{O}C_{PSS_{1}}^{S_{2}}{\left({\left[T\right]}_{S_{2}}^{O}\right)}^{T}\\ C_{PSS_{i}}^{O}=[T_{R,(i-1)\pi /2}](C_{PSS_{1}}^{O}){\left[T_{R,(i-1)\pi /2}\right]}^{T} \end{array}\right.$$

where

(10) $$\left\{\begin{array}{c} {\left[T\right]}_{S_{2}}^{O}=\left[\begin{array}{cc} {\left[R\right]}_{S_{2}}^{O} & 0_{3\times 3}\\ {\left[D\right]}_{S_{2}}^{O}{\left[R\right]}_{S_{2}}^{O} & {\left[R\right]}_{S_{2}}^{O} \end{array}\right],{\left[R\right]}_{S_{2}}^{O}=\left[R_{z,-\theta_{1}-\pi /2}\right]\left[R_{y,-\theta_{2}}\right],{\left[d\right]}_{S_{2}}^{O}=\left[\begin{array}{c} -l\sin\theta_{1}\cos\theta_{2}\\ R-l\cos\theta_{1}\cos\theta_{2}\\ -h_{1} \end{array}\right]\\ \theta_{1}=\arccos\left(\frac{R^{2}+l^{2}-{\left(h_{0}+h\right)}^{2}-r^{2}}{2R\sqrt{l^{2}-{\left(h_{0}+h\right)}^{2}}}\right),\;\theta_{2}=\arcsin\left(\frac{h_{0}+h}{l}\right) \end{array}\right.$$

According to Equation (6), the compliance matrix $C_{S_{3}}^{O}$ of the S branch in the global coordinate system {*O*} can be expressed as

(11) $$C_{S_{3}}^{O}={\left[T\right]}_{S_{3}}^{O}C_{S_{3}}{\left({\left[T\right]}_{S_{3}}^{O}\right)}^{T}$$

where

(12) $$\left\{{\left[T\right]}_{S_{3}}^{O}=\left[\begin{array}{cc} {\left[R\right]}_{S_{3}}^{O} & 0_{3\times 3}\\ {\left[D\right]}_{S_{3}}^{O}{\left[R\right]}_{S_{3}}^{O} & {\left[R\right]}_{S_{3}}^{O} \end{array}\right],\;{\left[R\right]}_{S_{3}}^{O}=\left[R_{z,-\pi /2}\right]\left[R_{y,-\pi /2}\right],\;{\left[d\right]}_{S_{3}}^{O}=\left[\begin{array}{c} 0\\ 0\\ -\left(h_{0}+h_{1}\right) \end{array}\right]\right.$$

In summary, after superimposing the compliances, the overall compliance matrix ***C****_T_* of the redundant-actuated 4-PSS&S compliant parallel mechanism in the global coordinate system {*O*} is

(13) $$C_{T}={\left({\left(C_{S_{3}}^{O}\right)}^{-1}+{\left(\sum_{i=1}^{4}C_{PSS_{i}}^{O}\right)}^{-1}\right)}^{-1}$$

### 3.2. Kinetostatic Modeling

The relationship between force and displacement at the center point of the mobile platform is obtained by the compliance model established in the previous section. However, the input and output of the compliant mechanism are usually not in the same coordinate system. Therefore, it is necessary to further explore the mapping relationship between input and output when the load is applied in different coordinate systems. Considering that a VCM is a DC servo motor that converts electrical signals into linear displacement based on the principle of Ampere’s force, its output is usually in the form of force or displacement. Thus, two kinetostatics models of the mechanism are constructed by taking force and displacement as the input forms of the mechanism, respectively.

**Figure 4:**
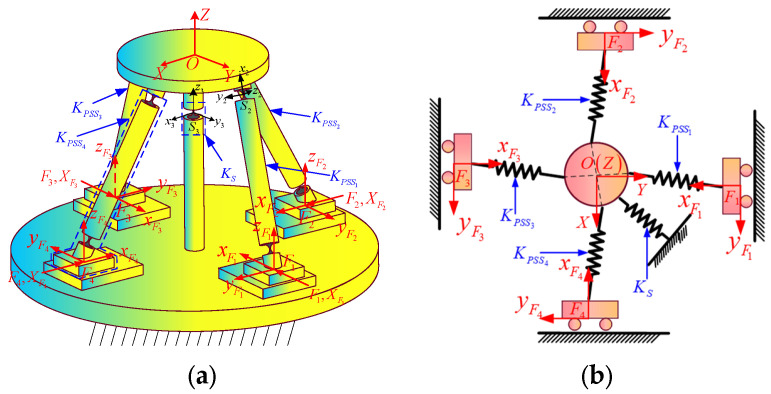
(**a**) Mechanism with force coordinate frames; (**b**) equivalent stiffness matrix of each branch.

In this section, the global coordinate system {*O*} is the same as that in Section 3.1.2. As shown in Figure 4a, the force coordinate systems $F_{i}-x_{i}y_{i}z_{i}$ (denoted as $\left\{F_{i}\right\}$, *i* = 1, 2, 3, 4) are established at the centers of the bottom surfaces of the four sliders, respectively. The corresponding generalized input forces and generalized input displacements are defined as $F_{i}={\left[m_{xi},m_{yi},m_{zi},f_{xi},f_{yi},f_{zi}\right]}^{T}$ and $X_{F_{i}}={\left[\theta_{xi},\theta_{yi},\theta_{zi},\delta_{xi},\delta_{yi},\delta_{zi}\right]}^{T}$, respectively. The displacement of the center point *O* of the mobile platform relative to its initial position in the coordinate system {*O*} caused by the input forces (or displacements) is $X_{O}={\left[\theta_{x},\theta_{y},\theta_{z},\delta_{x},\delta_{y},\delta_{z}\right]}^{T}$. For the convenience of analysis, the mechanism is equivalent to a spring system. The equivalent stiffness matrix of each branch is shown in Figure 4b. $K_{PSS_{i}}\left(i=1,2,3,4\right)$ and $K_{S}$ represent the equivalent stiffness matrices of the PSS*_i_* branch and the S branch, respectively.

#### 3.2.1. Establishment of Input/Output Force–Displacement Model (F-D Model)

**Figure 5:**
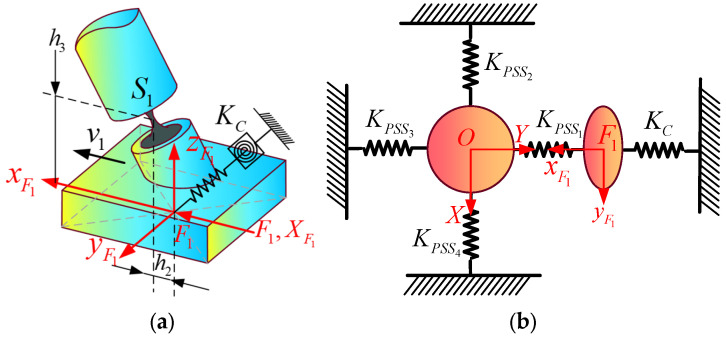
(**a**) Positional parameter of force coordinate system; (**b**) equivalent spring system model under ***F***_1_.

When the VCM is actuated in the form of force, only the acting force *f_xi_* along the moving direction of the slider in the generalized input force ***F****_i_* is known. Since the displacements in the five non-functional directions of the slider input displacement $X_{F_{i}}$ are all zero, during theoretical modeling, springs (two pieces) with infinite stiffness and torsion springs (three pieces) with infinite stiffness can be applied in the non-moving directions of the slider, and a spring (one piece) with infinitesimal stiffness can be applied in the functional direction, to simulate the situation where the displacement of the slider in the non-moving directions is zero. It is assumed that the constraint stiffness matrix composed of the stiffnesses of these six springs is $K_{C}=diag\left(\begin{array}{c} K_{\theta x},K_{\theta y},K_{\theta z},K_{\delta x},K_{\delta y},K_{\delta z} \end{array}\right)$, where $K_{\delta x}$ is infinitesimal and the remaining values are infinite. When the mechanism is only subjected to a single input force ***F***_1_, the relative positional relationship between the force coordinate system {*F*_1_} and the hinge *S*_1_ in the initial state is shown in Figure 5a, and the schematic diagram of the equivalent spring system model of the mechanism is shown in Figure 5b.

According to Hooke’s law, the elastic deformation control equation of the mechanism is as follows:

(14) $$\underset{K}{\underset{︸}{\left[\begin{array}{cc} {\left(K_{OO}\right)}_{F_{1}} & K_{OF_{1}}\\ K_{F_{1}O} & K_{F_{1}F_{1}} \end{array}\right]}}\left[\begin{array}{c} X_{1}\\ X_{F_{1}} \end{array}\right]=\left[\begin{array}{c} F_{O}\\ F_{1} \end{array}\right]$$

where $X_{F_{1}}$ represents the displacement of the slider in its own force coordinate system {*F*_1_}, and ***F_O_*** represents the external load applied at the center point *O* of the mobile platform. The calculations of the four sub-stiffness matrices in the overall stiffness matrix ***K*** are as follows:

(15) $$\left\{\begin{array}{c} {\left(K_{OO}\right)}_{F_{1}}=\sum_{i=1}^{4}K_{PSS_{i}}+K_{S}\\ K_{F_{1}F_{1}}=K_{PSS_{1}}^{F_{1}}+K_{C}={\left({\left[T\right]}_{S_{2}}^{F_{1}}\right)}^{-T}[K_{PSS_{1}}]{\left({\left[T\right]}_{S_{2}}^{F_{1}}\right)}^{-1}+K_{C}\\ K_{OF_{1}}=-K_{PSS_{1}}^{O,F_{1}}={\left({\left[T\right]}_{S_{2}}^{O}\right)}^{-T}[K_{PSS_{1}}]{\left({\left[T\right]}_{S_{2}}^{F_{1}}\right)}^{-1}\\ K_{F_{1}O}=-K_{PSS_{1}}^{F_{1},O}={\left({\left[T\right]}_{S_{2}}^{F_{1}}\right)}^{-T}[K_{PSS_{1}}]{\left({\left[T\right]}_{S_{2}}^{O}\right)}^{-1} \end{array}\right.$$

where

(16) $$\left\{\begin{array}{c} {\left[T\right]}_{S_{2}}^{F_{1}}=\left[\begin{array}{cc} {\left[R\right]}_{S_{2}}^{F_{1}} & 0_{3\times 3}\\ {\left[D\right]}_{S_{2}}^{F_{1}}{\left[R\right]}_{S_{2}}^{F_{1}} & {\left[R\right]}_{S_{2}}^{F_{1}} \end{array}\right],\;{\left[d\right]}_{S_{2}}^{F_{1}}=\left[\begin{array}{c} l\cos\theta_{1}\cos\theta_{2}+h_{2}\\ -l\sin\theta_{1}\cos\theta_{2}\\ l\sin\theta_{2}+h_{3} \end{array}\right],\;{\left[R\right]}_{S_{2}}^{F_{1}}=\left[R_{z,-\theta_{1}}\right]\left[R_{y,-\theta_{2}}\right]\\ K_{PSS_{i}}=C_{PSS_{i}}^{O},\;K_{S}=C_{S_{3}}^{O} \end{array}\right.$$

where the stiffness matrices $K_{PSS_{1}}^{F_{1}}$ with a single superscript *F*_1_ indicate that the corresponding force and displacement are both in the force coordinate system {*F*_1_}, while the stiffness matrices $K_{PSS_{1}}^{F_{1},O}$ with a double superscript (*F*_1_ and *O*) indicate that the corresponding force and displacement are located in the coordinate systems {*F*_1_} and {*O*}, respectively.

Assume that the mobile platform is not subjected to external forces; then, ***F_O_*** = **0**. Substituting it into Equation (14) gives

(17) $$X_{1}=\underset{C_{FX_{1}}}{\underset{︸}{-{\left({\left(K_{OO}\right)}_{F_{1}}-K_{OF_{1}}K_{F_{1}F_{1}}^{-1}K_{F_{1}O}\right)}^{-1}\left(K_{OF_{1}}K_{F_{1}F_{1}}^{-1}\right)}}\cdot F_{1}$$

The above formula describes the mapping relationship between the input force ***F***_1_ and the input displacement $X_{F_{1}}$, and $C_{FX_{1}}$ is the input compliance. Similarly, when the input force $F_{i}\left(i=2,3,4\right)$ is applied individually, the output displacement of the mobile platform can be expressed as

(18) $$X_{i}=\underset{C_{FX_{i}}}{\underset{︸}{-{\left({\left(K_{OO}\right)}_{F_{i}}-K_{OF_{i}}K_{F_{i}F_{i}}^{-1}K_{F_{i}O}\right)}^{-1}\left(K_{OF_{i}}K_{F_{i}F_{i}}^{-1}\right)}}\cdot F_{i}$$

Since the four PSS branches are uniformly distributed in the circumferential direction, each of the stiffness matrices ${\left(K_{OO}\right)}_{F_{i}}$, $K_{OF_{i}}$, $K_{F_{i}O}$ and $K_{F_{i}F_{i}}$ in Equation (18) can be directly obtained by rotating the corresponding stiffness matrix in $C_{FX_{1}}$ around the *Z*-axis of the global coordinate system {*O*} by 90°, 180° and 270°, respectively:

(19) $$\left\{\begin{array}{c} {\left(K_{OO}\right)}_{F_{i}}=\left[T_{R,\left(i-1\right)\pi /2}\right]\left[{\left(K_{OO}\right)}_{F_{1}}\right]{\left(\left[T_{R,\left(i-1\right)\pi /2}\right]\right)}^{T}\\ K_{F_{2}F_{2}}=K_{F_{3}F_{3}}=K_{F_{4}F_{4}}=K_{F_{1}F_{1}}\\ K_{OF_{i}}=\left[T_{R,\left(i-1\right)\pi /2}\right]\left[K_{OF_{1}}\right]\\ K_{F_{i}O}=\left[K_{F_{1}O}\right]{\left(\left[T_{R,\left(i-1\right)\pi /2}\right]\right)}^{T} \end{array}\right.$$

where

(20) $$\left[T_{R,\left(i-1\right)\pi /2}\right]=\left[\begin{array}{cc} R_{z,\left(i-1\right)\pi /2} & 0_{3\times 3}\\ 0_{3\times 3} & R_{z,\left(i-1\right)\pi /2} \end{array}\right]$$

Based on the superposition principle, the output displacements corresponding to the four input forces ***F****_i_* are superimposed to obtain the total output displacement ***X****_Total_* of the center point of the mobile platform, and its expression is as follows:

(21) $$X_{Total}=\sum_{i=1}^{4}X_{i}=\left[\begin{array}{cccc} C_{FX_{1}} & C_{FX_{2}} & C_{FX_{3}} & C_{FX_{4}} \end{array}\right]\cdot \left[\begin{array}{c} F_{1}\\ F_{2}\\ F_{3}\\ F_{4} \end{array}\right]$$

For the convenience of practical application, the relationship between the input force ***F****_i_* along the moving direction of the slider and the angular displacements ***θ****_Total_* in the three degree-of-freedom directions of the mobile platform can be further extracted from Equation (21). Therefore, the input/output force–displacement model of the redundant-actuated 4-PSS&S compliant parallel mechanism can be expressed by the following equation:

(22) $$\theta_{Total}=\left[\begin{array}{c} \theta_{x}\\ \theta_{y}\\ \theta_{z} \end{array}\right]=\left[\begin{array}{cccc} {\left(C_{FX_{1}}\right)}_{\left[1\sim 3,4\right]} & {\left(C_{FX_{2}}\right)}_{\left[1\sim 3,4\right]} & {\left(C_{FX_{3}}\right)}_{\left[1\sim 3,4\right]} & {\left(C_{FX_{4}}\right)}_{\left[1\sim 3,4\right]} \end{array}\right]\cdot \left[\begin{array}{c} f_{x1}\\ f_{x2}\\ f_{x3}\\ f_{x4} \end{array}\right]$$

where ${\left(C_{FX_{i}}\right)}_{\left[1\sim 3,4\right]}$ represents the first three elements of the fourth column of the mapping matrix $C_{FX_{i}}$.

It is worth noting that Equation (22) is an underdetermined system of equations. When a set of outputs is given, theoretically, there is an infinite number of solutions for the input forces. This provides the possibility for the optimal selection of the peak actuating force.

#### 3.2.2. Establishment of Input/Output Displacement–Displacement Model (D-D Model)

When the VCM is actuated in the form of displacement, it is assumed that the input displacement of the slider is $X_{F_{i}}={\left[0,0,0,\delta_{xi},0,0\right]}^{T}$. At this moment, it is only necessary to replace the constraint stiffness matrix $K_{C}$ in Equations (14) and (15) with a 6 × 6 zero matrix. Then, according to Equations (18) and (21), the relationship between the input displacement $X_{F_{i}}$ and the output displacement $X_{i}$ can be derived. Furthermore, the total output displacement ***X****_Total_* of the mechanism is obtained as follows:

(23) $$X_{Total}=\sum_{i=1}^{4}X_{i}=\left[\begin{array}{cccc} C_{XX_{1}} & C_{XX_{2}} & C_{XX_{3}} & C_{XX_{4}} \end{array}\right]\cdot \left[\begin{array}{c} X_{F_{1}}\\ X_{F_{2}}\\ X_{F_{3}}\\ X_{F_{4}} \end{array}\right]$$

where

(24) $$C_{XX_{i}}=-{\left({\left(K_{OO}\right)}_{f_{i}}-K_{OF_{i}}K_{F_{i}F_{i}}^{-1}K_{F_{i}O}\right)}^{-1}\left(K_{OF_{i}}K_{F_{i}F_{i}}^{-1}\right)\left(K_{F_{i}F_{i}}-K_{F_{i}O}{\left(K_{OO}\right)}_{f_{i}}^{-1}K_{OF_{i}}\right)$$

Thus, the input/output displacement–displacement model of the redundant-actuated 4-PSS&S compliant parallel mechanism is as follows:

(25) $$\theta_{Total}=\left[\begin{array}{c} \theta_{x}\\ \theta_{y}\\ \theta_{z} \end{array}\right]=\left[\begin{array}{cccc} {\left(C_{XX_{1}}\right)}_{\left[1\sim 3,4\right]} & {\left(C_{XX_{2}}\right)}_{\left[1\sim 3,4\right]} & {\left(C_{XX_{3}}\right)}_{\left[1\sim 3,4\right]} & {\left(C_{XX_{4}}\right)}_{\left[1\sim 3,4\right]} \end{array}\right]\cdot \left[\begin{array}{c} \delta_{x1}\\ \delta_{x2}\\ \delta_{x3}\\ \delta_{x4} \end{array}\right]$$

### 3.3. Example Verification

In this paper, the Static Structural module in the software ANSYS-Workbench 19.2 is used to verify the two kinetostatic models. The specific steps are as follows: Firstly, obtain the target pose trajectory of the center point of the mobile platform and discretize it into several sample points. Substitute these sample points into Equations (22) and (25), respectively, and use the pseudo-inverse algorithm [13] to obtain the theoretical input force and input displacement, respectively. Then, the theoretical input results obtained are applied to the finite element model to obtain two simulated pose trajectories. Through a comparison of these two trajectories with the target pose trajectory, respectively, the correctness of the two models can be verified. In this example, 65Mn is selected as the material of the flexible spherical hinge. Its Young’s modulus is 206 GPa, density is 7850 kg/m^3^ and Poisson’s ratio is 0.3. For the remaining structures with relatively high stiffness, structural steel is selected as the material. The geometric parameters related to the mechanism and the flexible spherical hinge are shown in Table 1.

The specified target pose trajectory of the mobile platform is as follows:

(26) $$\left\{\begin{array}{l} \alpha =R_{1}\sin\theta \cos p\theta \\ \beta =R_{1}\sin\theta \sin p\theta \\ \gamma =R_{1}\cos\theta \\ R_{1}=0.02rad,0\leq \theta \leq \pi ,p=10 \end{array}\right.$$

**Figure 6:**
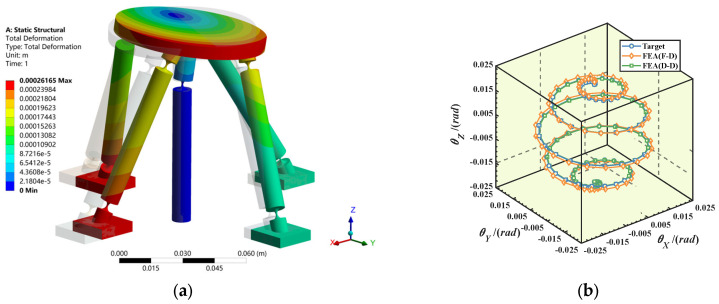
(**a**) Finite element simulation cloud image; (**b**) comparison of target and simulation trajectory.

To make the simulation results more accurate, for the area of flexible materials where deformation is relatively concentrated, hexahedral meshes with a size of 0.3 mm are adopted to improve the accuracy of the stress field solution. For the remaining structures with relatively high stiffness, tetrahedral meshes of 3 mm are used for discretization. The obtained finite element simulation cloud image is shown in Figure 6a. The comparison diagrams of the target trajectories and simulation trajectories of the two kinetostatic models are shown in Figure 6b.

**Figure 7:**
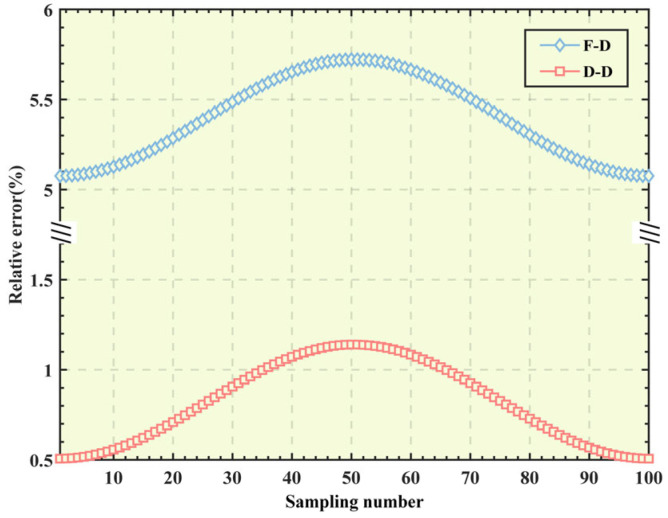
Relative error of two mapping models.

Figure 7 shows the relative errors of the two models. The results indicate that the relative error of the input/output force–displacement model is less than 6%, while the relative error of the input/output displacement–displacement model is relatively small, within 1.5%. The good consistency of the trajectories demonstrates that both of the established kinetostatic models are correct, and the laws of the changes in the errors of the two models with the sampling points are the same.

## 4. Comparative Analysis of Mechanism Performance

**Figure 8:**
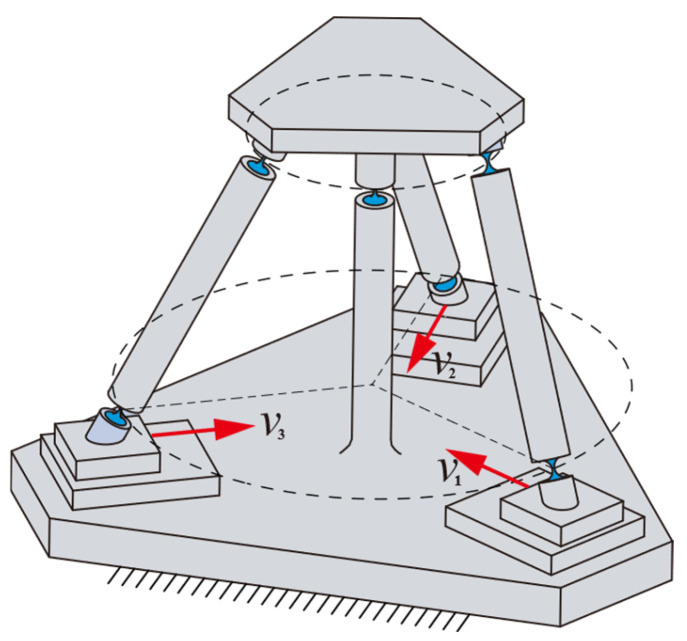
Axonometric drawing of 3-PSS&S.

In the previous section, the kinetostatic model of the redundant-actuated 4-PSS&S compliant parallel mechanism was established. In this section, the performance of this mechanism including the peak actuating force, output stiffness, parasitic axis drift and workspace will be further analyzed. In order to explore the effect of redundant actuation on improvement in the mechanism’s performance, the performance of the 3-PSS&S mechanism (the axonometric drawing is shown in Figure 8) and the non-redundant-actuated 4-PSS&S mechanism (that is, no input force exerted on the fourth branch) is also analyzed, and a comparison is made with the performance of the redundant-actuated 4-PSS&S mechanism. It should be noted that the mechanism parameters selected in this section and the target trajectory used in the simulation are consistent with the parameters in Table 1 of Section 3.3 and the trajectory corresponding to Equation (26), respectively.

### 4.1. Comparative Analysis of Peak Actuating Force

Discretize the target pose trajectory into 100 sample points, and substitute them into the input/output force–displacement and input/output displacement–displacement models in Equations (22) and (25) in sequence. Through the use of the pseudo-inverse algorithm, 100 sets of input force solutions and input displacement solutions can be obtained, respectively. Among them, the peak actuating force and the peak actuating displacement are 7.146 N and 1.303 mm, respectively. Since the peak output force of the VCM selected in Section 2 is 6.8 N and the total stroke is 4 mm, the output displacement of the VCM is sufficient at this time, but the output force cannot meet the requirements of the actuating force needed by the mechanism. Therefore, when solving the input force in Equation (22), the peak actuating force required by the mechanism is expected to be reduced by optimizing the actuating force distribution. Since Equation (22) is a four-input, three-output model, given a set of output angular displacements, there are infinitely many sets of input force solutions. The parameters of the redundant-actuated 4-PSS&S mechanism are substituted into Equation (22), and the result form of the input/output force–displacement mapping matrix is as shown in Equation (27). In this context, *a*, *b* and *c* are constants.

(27) $$C_{FX}=\left[\begin{array}{cccc} a & -b & -a & b\\ b & a & -b & -a\\ c & c & c & c \end{array}\right]$$

In observing the mapping matrix $C_{FX}$, it is not difficult to find that there is a constant particular solution $X_{0}={\left[\begin{array}{cccc} -1 & 1 & -1 & 1 \end{array}\right]}^{T}$ in its null space. Therefore, the input force solution $F_{in}$ of the redundant-actuated 4-PSS&S mechanism can be expressed as

(28) $$F_{in}=\left[\begin{array}{c} f_{1}\\ f_{2}\\ f_{3}\\ f_{4} \end{array}\right]=\left[\begin{array}{c} f_{1}^{\prime }\\ f_{2}^{\prime }\\ f_{3}^{\prime }\\ 0 \end{array}\right]+\omega \cdot \left[\begin{array}{c} -1\\ 1\\ -1\\ 1 \end{array}\right]$$

where *ω* is the force distribution coefficient. It is worth noting that when *ω* = 0, the obtained solution corresponds to the input force solution of the non-redundant-actuated 4-PSS&S mechanism.

**Figure 9:**
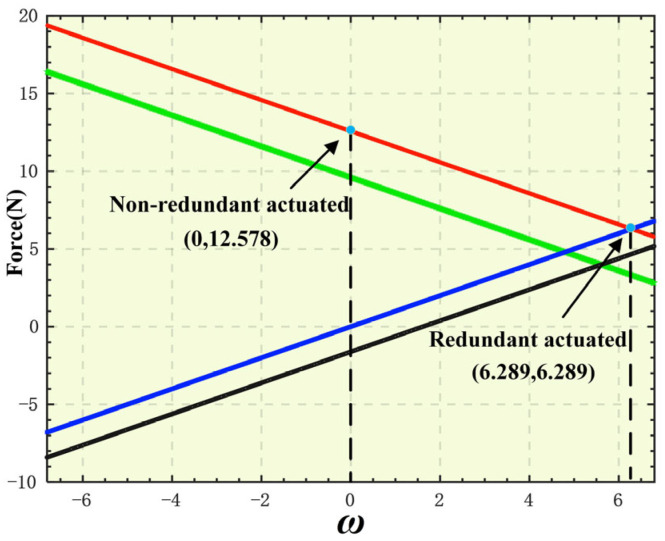
Influence of force distribution coefficient *ω* on input force of other branches.

Taking the input force range [−6.8 N, 6.8 N] of the selected VCM as the constraint condition, by varying the force distribution coefficient *ω*, a set of solutions with the minimum peak actuating force can be selected. Select a point [−5.843 × 10^−3^ rad, −4.595 × 10^−3^ rad, 1.857 × 10^−3^ rad] from the target spherical trajectory and substitute it into Equation (22) for solving. A solution in the form of Equation (28) is obtained. The influence of the force distribution coefficient *ω* on the input force solution is shown in Figure 9. As can be seen from Figure 9, when *ω* = 0, the input force solution of the non-redundant-actuated 4-PSS&S mechanism is [9.600 N, −1.625 N, 12.578 N]^T^. At this time, the required peak actuating force is 12.578 N, which exceeds the maximum output force of 6.8 N of the selected VCM. It can be seen that the non-redundant-actuated mode cannot meet the driving requirements. When *ω* = 6.289, the input force solution of the redundant-actuated 4-PSS&S mechanism is [3.311 N, 4.674 N, 6.289 N, 6.289 N]^T^. At this time, the required peak actuating force is 6.289 N, and the maximum output force of 6.8 N of the selected VCM can meet the requirements. This result indicates that by applying redundant actuation, the peak actuating force required by the 4-PSS&S mechanism can be effectively reduced.

**Figure 10:**
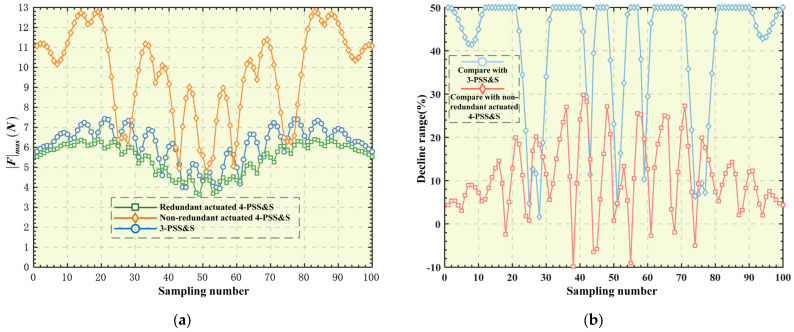
(**a**) Peak force of different mechanisms; (**b**) decline range on trajectory.

Similarly, following the above steps, the optimal peak actuating force corresponding to other points on the trajectory can be solved in sequence. For the convenience of comparison, the peak actuating force of the 3-PSS&S mechanism under the target trajectory is also calculated. The comparison results of the peak actuating force of the three mechanisms are shown in Figure 10a. The results show the following: ① After the optimal distribution of the actuating force of the redundant-actuated 4-PSS&S mechanism, its peak actuating force decreases significantly compared with the non-redundant-actuated 4-PSS&S mechanism. The corresponding reduction amplitude of each point on the trajectory is shown in Figure 10b; the maximum reduction amplitude can reach 50%, with the average reduction amplitude being 40.95%. This indicates that introducing redundant actuation combined with the optimal distribution of actuating forces can effectively reduce the peak actuating force of the 4-PSS&S mechanism. ② Compared with the 3-PSS&S mechanism, the reduction amplitude of the peak force of the redundant-actuated 4-PSS&S mechanism is slightly smaller (there is even a slight increase at a few target points), and there is an average reduction of 10.79% in general. This is because the 4-PSS&S mechanism has greater structural stiffness than the 3-PSS&S mechanism, which to some extent increases the actuating force of the 4-PSS&S mechanism.

### 4.2. Comparison of Output Stiffness

The output stiffness is an important index for evaluating the ability of a parallel mechanism to resist deformation caused by external loads, and it has a direct relationship with the bearing capacity of the mechanism. In the field of dynamics, the output stiffness is also positively correlated with the response speed and natural frequency of the mechanism [14]. In this section, a comparative analysis is carried out of the output stiffness of the redundant-actuated 4-PSS&S mechanism and the 3-PSS&S mechanism in the three main functional directions. The overall compliance of the 3-PSS&S mechanism has been given in Ref. [11]. Just by inverting it, the output stiffness matrix $K_{output}$ of the mechanism can be obtained. For the redundant-actuated 4-PSS&S mechanism, it can be directly obtained by inverting the overall compliance matrix $C_{T}$ of the mechanism according to Equation (13):

(29) $$K_{output}={\left(C_{T}\right)}^{-1}$$

Substitute the mechanism parameters in Table 1 into the output stiffness matrices of the 3-PSS&S mechanism and 4-PSS&S mechanism, respectively. The first three elements on the diagonal are the output stiffnesses in the directions of *θ_x_*(*θ_y_*) and *θ_z_*. The comparison results of the output stiffnesses of the two mechanisms are shown in Table 2. The results show the following: ① The output stiffnesses of the two mechanisms in the *X* and *Y* directions are the same, which is due to the symmetry of the mechanism in the *XOY* plane. ② Compared with the 3-PSS&S mechanism, the output stiffnesses of the redundant-actuated 4-PSS&S mechanism in the *θ_x_* and *θ_y_* directions increase by 26.68%, and that in the *θ_z_* direction increases by 33.31%. This indicates that the increase in redundant branches can significantly improve the output stiffness of the mechanism.

### 4.3. Comparative Analysis of Parasitic Axis Drift

**Figure 11:**
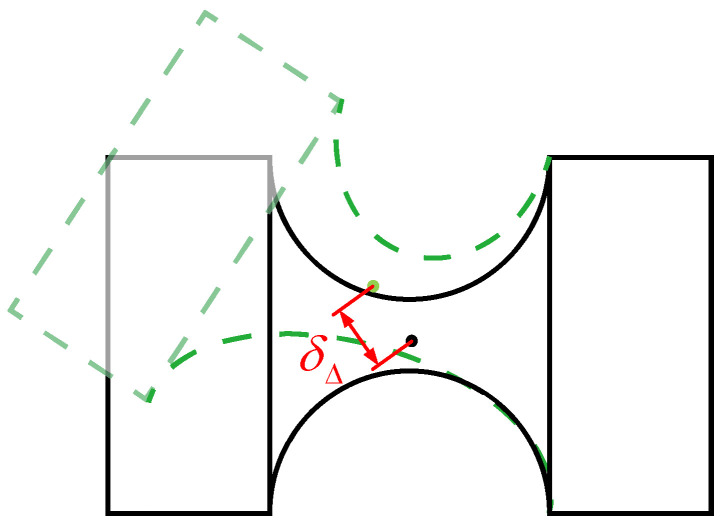
A diagram of parasitic axis drift.

During the motion process of the compliant hinge, the phenomenon of parasitic axis drift (PAD) will inevitably occur; that is, the rotation center is not constant but shifts with the change in the rotation angle, and it will increase as the rotation angle increases [15]. Its schematic diagram is shown in Figure 11, and *δ_Δ_* represents the displacement of PAD. Reducing the PAD of the compliant hinge is of great significance for improving the motion accuracy of the mechanism. In the 4-PSS&S compliant parallel mechanism, the rotation range of the restraining spherical hinge *S*_3_ is much larger than that of other compliant spherical hinges, which leads to a larger PAD of the spherical hinge *S*_3_. Therefore, the PAD of the restraining spherical hinge *S*_3_ will mainly be analyzed below.

**Figure 12:**
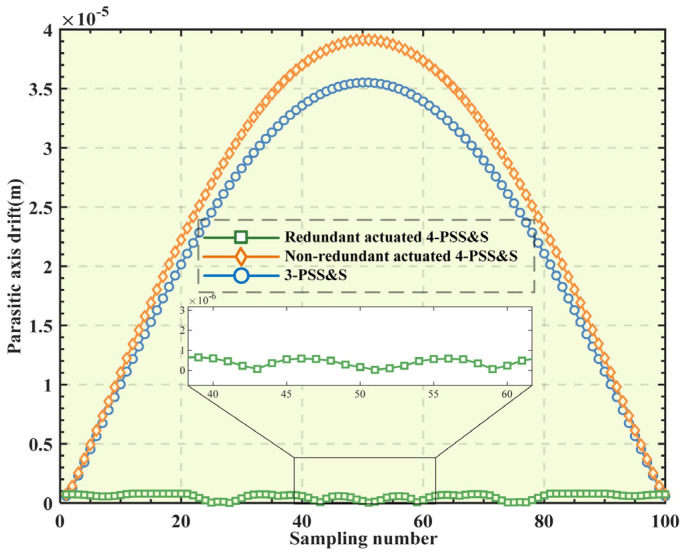
A comparison of parasitic axis drift.

Based on the mechanism parameters in Table 1 and the physical parameters in Section 3.3, the finite element models of the three mechanisms (the redundant-actuated 4-PSS&S mechanism, the non-redundant-actuated 4-PSS&S mechanism and the 3-PSS&S mechanism) are constructed, respectively. The target trajectory chosen is still the spherical trajectory corresponding to Equation (26) Then, the finite element simulation is performed to obtain the PAD conditions of the restraining spherical hinge *S*_3_ of the three mechanisms. The specific process is as follows: Discretize the target trajectory into 100 sample points, and substitute them into the kinetostatic models of the three mechanisms in sequence to solve the corresponding input forces. Then, input the obtained input force into the finite element model to obtain the PAD displacement of the restraining spherical hinge *S*_3_. The obtained results are sorted as shown in Figure 12. It is not difficult to see that the PAD displacement generated by the restraining spherical hinge of the redundant-actuated 4-PSS&S mechanism is much smaller than that of the non-redundant-actuated 4-PSS&S mechanism and the 3-PSS&S mechanism. The reasons for this are as follows: Firstly, the uniform distribution of the four branches of the redundant-actuated 4-PSS&S mechanism gives it orthogonality (that is, the movement directions of the sliders intersect at a right angle). Secondly, after introducing redundant actuation and the optimal distribution of actuating force (with the goal of minimizing the peak force), the difference in the input forces exerted on each driving branch becomes smaller. This makes the rotation range of the restraining spherical hinge *S*_3_ smaller when the platform reaches the same pose, thereby reducing the PAD of the spherical hinge.

### 4.4. Analysis of Kinetostatic Workspace

The workspace of the mechanism [16] refers to the set of spatial points that the end-effector of the mechanism can reach. Due to “inherent defects” such as the small deformation of the compliant units and the relatively small workspace of the parallel mechanism itself, it is of great significance to increase the workspace of the compliant parallel mechanism. In this section, based on the kinetostatic model, taking the limit rotation angle of the restraining spherical hinge *S*_3_, the peak actuating force and the peak actuating displacement of the VCM as the constraint conditions, the limit boundary numerical search algorithm [17] is used to solve the workspace of the mechanism. Since the rotation of the mobile platform is consistent with the rotation of the restraining spherical hinge *S*_3_, the limit rotation amplitude of the compliant rotation angle is restricted by limiting the motion range of the mobile platform.

**Figure 13:**
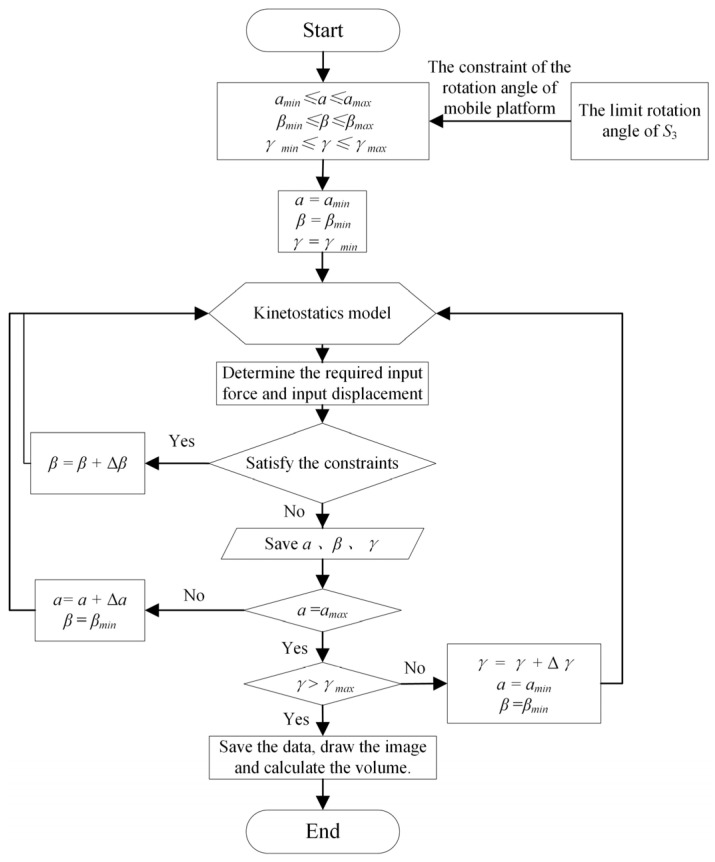
The flowchart of solving the workspace.

The specific solving process of the workspace is as follows: First, based on the finite element model of the compliant hinge, combined with the ultimate stress of the material, the ultimate inclination angle and ultimate torsion angle of the compliant hinge can be obtained, which are 1.6° and 1.3°, respectively. On this basis, the pose range of the mobile platform is set as 0~360° × 0~1.6° × ±1.3°. Then, the space is discretized into several points, and a search is carried out from bottom to top to calculate the required input force and input displacement under each pose. The limit input force of the selected VCM is 6.8 N and the input displacement is 4 mm. In order to ensure the optimal working performance of the VCM, the limit input force *F_max_* = 6 N and the limit input displacement *X_max_* = 3 mm are taken, and the pose points that meet the requirements are screened out. The set of all the pose points thus formed is the workspace of the mechanism. The detailed flowchart is shown in Figure 13.

**Figure 14:**
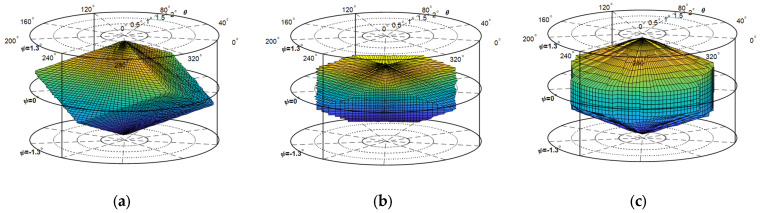
The workspace of each mechanism. (**a**) 3-PSS&S mechanism. (**b**) Non-redundant-actuated 4-PSS&S mechanism. (**c**) Redundant-actuated 4-PSS&S mechanism.

Considering that the three degrees of freedom of the mechanism are all rotational, it is more intuitive to use the cylindrical coordinate system to represent the workspace. The workspaces of the 3-PSS&S mechanism, the non-redundant-actuated 4-PSS&S mechanism and the redundant-actuated 4-PSS&S mechanism are shown in Figure 14a–c, respectively. In this context, the polar angle, polar radius and height of the coordinate system represent the mechanism pose angles *a*, *β* and *γ*, respectively. As shown in Figure 14a, the workspace of the 3-PSS&S mechanism is shaped like a rhombohedral body with a three-fold symmetry distribution in space. Its six surfaces are all rhombuses and of similar sizes. The workspace of the non-redundant-actuated 4-PSS&S mechanism, as shown in Figure 14b, has an irregular shape, resembling a rhombohedral body with its top and bottom corners cut off. The workspace of the redundant-actuated 4-PSS&S mechanism, as shown in Figure 14c, is shaped like a geometric body with a four-fold symmetry distribution in space, and its shape is similar to the combination of an octahedral structure intersecting with a cylinder. It can be seen from Figure 14a,c that the workspaces of the 3-PSS&S mechanism and the redundant-actuated 4-PSS&S mechanism are symmetric, which is because the actuation of these two mechanisms is symmetric.

The calculation results of the equivalent workspace volumes (in radian system) of the three mechanisms in the Cartesian coordinate system show that the equivalent workspace volumes of the 3-PSS&S mechanism, the non-redundant-actuated 4-PSS&S mechanism and the redundant-actuated 4-PSS&S mechanism are 1.413 × 10^−5^, 5.804 × 10^−6^ and 2.745 × 10^−5^, respectively. The results indicate that after the optimal distribution of the actuating force for the redundant-actuated 4-PSS&S mechanism, its workspace volume has increased by 94.32% compared with the 3-PSS&S mechanism and has increased by 372.89% compared with the non-redundant-actuated 4-PSS&S mechanism.

## 5. Conclusions

This paper proposes a novel redundant-actuated 4-PSS&S compliant parallel 3-DOF micro-rotation mechanism and establishes the kinetostatic model of this mechanism. Then, the performance of this mechanism, such as peak actuating force, output stiffness, parasitic axis drift and workspace, is analyzed and compared with the similar 3-PSS&S mechanism and the non-redundant-actuated 4-PSS&S mechanism. The conclusions are as follows:

1. For the 4-PSS&S mechanism, redundant actuation combined with optimized actuating force distribution can effectively reduce the peak actuating force, with the maximum reduction rate reaching up to 50% and the average reduction amplitude being 40.95%. Compared with the 3-PSS&S mechanism, the reduction rate of the peak force of the redundant-actuated 4-PSS&S mechanism is slightly smaller, with an average reduction rate of 10.79% in general.
2. Compared with the 3-PSS&S mechanism, the output stiffness of the 4-PSS&S mechanism increases by 26.68% in the *θ_x_* and *θ_y_* directions and by 33.31% in the *θ_z_* direction, indicating that the addition of a redundant branch can significantly improve the output stiffness of the mechanism.
3. Compared with the 3-PSS&S mechanism and the non-redundant-actuated 4-PSS&S mechanism, after the optimal distribution of the actuating force, the parasitic axis drift of the redundant-actuated 4-PSS&S mechanism at the restraining spherical hinge *S*_3_ is significantly reduced. This shows that the redundant-actuated 4-PSS&S mechanism has higher motion accuracy compared with the other two mechanisms.
4. After the optimal distribution of the actuating force, the volume of the workspace of the redundant-actuated 4-PSS&S mechanism increases by 94.32% compared with that of the 3-PSS&S mechanism and by 372.89% compared with that of the non-redundant-actuated 4-PSS&S mechanism.

In future research, the impact of redundant actuation on the dynamic performance of the mechanism will continue to be explored. At the same time, other advanced control strategies will be applied to the mechanism to further improve its related performance.

## Figures and Tables

**Table 1:** The fundamental parameters of the mechanism.

Parameter	Value (mm)	Parameter	Value (mm)
*r*	30	*r*_0_	2.5
*R*	60	*t_min_*	0.8
*h*_0_	15	*h*_1_	13
*h*	50	*h*_2_	2
*l*	72.5	*h*_3_	12

**Table 2:** A comparison of the output stiffness.

Output Stiffness (N/rad)	3-PSS&S	4-PSS&S	Increase in Amplitude
*θ_x_*(*θ_y_*)	2.353 × 10^4^	2.981 × 10^4^	+26.68%
*θ_z_*	3.414 × 10^3^	4.552 × 10^3^	+33.31%

## Data Availability

All data that support the findings of this study are included within the article.

## Appendix A

The compliance coefficients of the right-circular flexure spherical hinge in the coordinate system at the center of its end face *O_B_* are as follows:

(A1) $$\left\{\begin{array}{l} C_{\theta_{x},m_{x}}=\frac{64\left(1+\nu \right)}{\pi E}\int_{0}^{2r_{0}}\frac{1}{t{\left(x\right)}^{4}}dx,\\ C_{\theta_{y},m_{y}}=C_{\theta_{z},m_{z}}=\frac{64}{\pi E}\int_{0}^{2r_{0}}\frac{1}{t{\left(x\right)}^{4}}dx,\\ C_{\delta_{z},m_{y}}=C_{\theta_{y},f_{z}}=-C_{\theta_{z},f_{y}}=-C_{\delta_{y},m_{z}}=-\frac{64r_{0}}{\pi E}\int_{0}^{2r_{0}}\frac{1}{t{\left(x\right)}^{4}}dx,\\ C_{\delta_{x},f_{x}}=\frac{4}{\pi E}\int_{0}^{2r_{0}}\frac{1}{t{\left(x\right)}^{2}}dx\\ C_{\delta_{y},f_{y}}=C_{\delta_{z},f_{z}}=\frac{64}{\pi E}\int_{0}^{2r_{0}}\frac{x^{2}}{t{\left(x\right)}^{4}}dx+\frac{k\left(1+v\right)}{\pi E}\int_{0}^{2r_{0}}\frac{1}{t{\left(x\right)}^{2}}dx, \end{array}\right.$$

where *E* and *ν* represent the Young’s modulus and Poisson’s ratio of the material of the flexure spherical hinge, respectively, *k* is the shear shape coefficient, and *k* = 10/9 is taken. *t*(x) is the thickness function of the flexure spherical hinge along the *x*-axis direction, and its definition is as follows:

(A2) $$t\left(x\right)=t_{\min}+2\left(r_{0}-\sqrt{x\left(2r_{0}-x\right)}\right)\;\;\;\left(x\in \left[0,2r_{0}\right]\right)$$
